# Engagement Book

**Published:** 1880-10

**Authors:** 


### ENGAGEMENT BOOK—EDITION FOR 1881.

There is in press, and will be issued in a few days, "*The Dentist's Memorandum; Book of Engagements; and Manual of Ready Reference for* 1881, *by Dr. J. Taft.*

This book, which was prized so highly by many in the profession for several years, has been out of print for the last six years.

At the request of quite a number who have used it, it has been decided to publish it for 1881.

It differs from all other such books, in the fact that it has the months, and days of the month through the year. It also has the hours of each day, from 8 A. M. to 6 P. M., for appointments.

It also contains a large number of formulas recipes, suggestions, and directions, all of which are useful to the dentist. It will be neatly gotten up, bound in two styles—cloth and morocco.

It will be on sale by the first of November.

